# Low Prevalence of *Toxoplasma gondii* in Sheep and Isolation of a Viable Strain from Edible Mutton from Central China

**DOI:** 10.3390/pathogens12060827

**Published:** 2023-06-14

**Authors:** Yibao Jiang, Shilin Xin, Yiheng Ma, Heng Zhang, Xu Yang, Yurong Yang

**Affiliations:** 1 College of Animal Science and Technology, Henan Agricultural University, Zhengzhou 450000, China; 2 College of Veterinary Medicine, Henan Agricultural University, Zhengzhou 450000, China

**Keywords:** *Toxoplasma gondii*, sheep, epidemiology, vertical transmission, isolation

## Abstract

Sheep are highly susceptible to *Toxoplasma gondii*, and miscarriage is the main clinical feature. This study investigated 227 sheep samples (210 myocardial tissues from slaughterhouses, 6 ewe serum samples, 3 aborted fetuses, and 8 dead lambs from veterinary clinics) from central China for *T. gondii* infection. Antibodies against *T. gondii* were detected using the modified agglutination test (MAT). PCR was performed to detect *T. gondii* DNA in the tissue samples. The results showed that four samples were seropositive (MAT titer ≥ 1:100), with a seroprevalence of 1.8% (4/227). The seropositive samples included two myocardial samples from a slaughterhouse, one ewe and its aborted fetus from a veterinary clinic. The results revealed that 7 out of 207 (3.4%) sheep tissue samples were PCR-positive, including two myocardial tissue samples from slaughterhouses, three aborted fetuses, and two lambs from veterinary clinics. *Toxoplasma gondii* vertical transmission had occurred in two of three pairs of ewes and her pups. One viable *T. gondii* strain (TgSheepCHn14) was isolated from the myocardial tissues of sheep from a slaughterhouse. Tachyzoites were obtained from cell cultures at 70 days following seeding in the brains and lungs of mice. This strain was non-lethal to Swiss mice. The number of parasite brain cysts in mice decreased with time post-infection (*p* < 0.05). Overall, the prevalence of *T. gondii* in the sheep samples was low. Although the samples were scattered, and not from planned collections, the current study detected *T. gondii* antibodies and DNA in aborted fetuses, indicating that vertical transmission could occur and maintain the parasites in sheep herds without exogenous infection.

## 1. Introduction

*Toxoplasma gondii* is an intracellular protozoan that infects all warm-blooded animals, including humans and livestock. Members of the felid family are the definitive hosts of *T. gondii*, while other warm-blooded animals may be intermediate hosts. Over the last decade, the prevalence of *T. gondii* in humans has decreased [1]. *Toxoplasma gondii* infection is usually asymptomatic or subclinical in immune-competent patients and animals. However, *T. gondii* infection during gestation may cause miscarriage, fetal death, and eye or brain disorders [2,3]. Sheep are highly susceptible to *T. gondii* infection. Studies have found that the mutton, viscera, blood, and milk of sheep or goats with acute toxoplasmosis can transmit the parasite to other animals and humans [4,5,6]. The seroprevalence of *T. gondii* in sheep (2000–2017) from China was 11.8% (2305/19,565), which was lower than that in other countries [7,8]. Abortion history and age are the main risk factors for *T. gondii* infection in sheep [8,9]. To date, at least 244 viable *T. gondii* strains have been isolated from the tissues (heart, brain, lung, diaphragm, and skeletal muscle) of sheep globally: 97% of strains from sheep in Europe and Africa are Type II (ToxoDB #1 or #3) [8]. However, only 14 viable *T. gondii* strains were recovered from sheep tissues in China, including ToxoDB #3 (n = 1), ToxoDB #9 (n = 2), ToxoDB #2 (n = 7), and ToxoDB #4 (n = 4) [9,10,11].

There are two main modes of transmission of *T. gondii*: horizontal and vertical transmission [12,13]. Vertical transmission has been reported in sheep in many countries, such as New Zealand, Australia, the United Kingdom, Norway, and the United States [2,14]. China has the largest number of sheep worldwide, with an estimated 164 million domestic sheep. However, research on *T. gondii* infection in sheep is limited. The objective of this study is to assess *T. gondii* infection and transplacental transmission in sheep, and to isolate viable *T. gondii* strains from mutton that is intended for human consumption.

## 2. Materials and Methods

### 2.1. Sample Collection and Background Information

**Figure 1:**
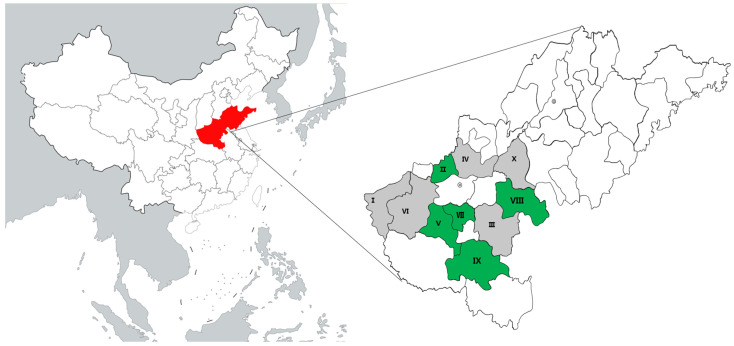
Map showing the location of samples received from the Henan and Shandong provinces in China. I: Sanmenxia; II: Jiaozuo; III: Zhoukou; IV: Xinxiang; V: Pingdingshan; VI: Luoyang; VII: Xuchang; VIII: Shangqiu; IX: Zhumadian; X: Heze. Green represents sheep samples from the areas that tested positive for *Toxoplasma gondii*.

From 2019 to 2021, 227 sheep samples (210 myocardial tissues from slaughterhouses, 6 ewe serum samples, 3 aborted fetuses, and 8 dead lambs from veterinary clinics) were collected from Henan and Shandong provinces (Table 1, Figure 1). All the samples were collected and transported to the Laboratory of Veterinary Pathology, Henan Agricultural University (Zhengzhou, Henan, China) for pathological diagnosis and evaluation of meat quality. This study also allowed us to survey *T. gondii* infections in these samples.

### 2.2. Detection of Antibodies against T. gondii in Sheep

All of the serum or heart fluid samples were tested for IgG antibodies against *T. gondii* using a modified agglutination test (MAT) [15]. MAT titers of 1:100 or higher were considered positive for *T. gondii*; negative and positive controls were included for every plate. Heart fluid titers of 1:100 or higher were considered positive for *T. gondii,* which was determined by comparing parasite isolation with MAT titers from sheep [10,16]. Whole formalin-treated *T. gondii* tachyzoites were obtained from the University of Tennessee Research Foundation (Knoxville, TN, USA; https://utrf.tennessee.edu/, accessed on 6 May 2019). Serum from *T. gondii* VEG-infected mice was provided by Dr. J. P. Dubey (Beltsville, ARS, USDA, USA), and it was used as a reference. Samples from veterinary clinics were tested for IgM antibodies against *T. gondii* using test papers (2206D014: Anigen Bionote, Seoul, Republic of Korea).

### 2.3. DNA Extraction and Genomic Detection of T. gondii in Sheep

DNA was extracted from tissue samples and pepsin-digested tissues of sheep with a commercial DNA extraction kit (Tiangen Biotec Company, Beijing, China, DP304). PCR was used to detect *T. gondii* DNA using specific primer pairs TOX5/TOX8 [17]. The length of the PCR product was estimated to be 450 bp, and both negative and positive controls (*T. gondii* VEG-infected mice) were used.

### 2.4. Histopathological Analysis

Tissue samples (myocardial tissues and tissues from fetuses or lambs) from all the sheep were fixed in 10% (*v*/*v*) neutral-buffered formalin. Tissues were processed using routine histological techniques and then embedded in paraffin. Paraffin sections (5 μm thick) of the samples were prepared and stained with hematoxylin and eosin (H&E). Tissues suspected of being infected with *T. gondii* were subjected to immunohistochemistry (IHC) staining. IHC was performed with rabbit anti-*T. gondii* serum as the primary antibody and mouse anti-rabbit IgG conjugated with HRP/DAB (ab64264; Abcam, Waltham, MA, USA) as the secondary antibody. Brain tissue sections from a VEG *T. gondii*-infected mouse (provided by Dr. Dubey; ARS, USDA) were used as the positive control for IHC.

### 2.5. Isolation of a Viable T. gondii from Sheep Myocardial Tissues Using Mouse Bioassay

Mouse bioassay was performed on four sheep myocardial tissue samples (Jiaozuo) (Table 1), according to published methods [2]. In brief, every myocardial tissue (50 g) was digested in a pepsin solution and subcutaneously inoculated into Swiss mice (n = 4) or a gamma interferon (IFN-γ) knockout mouse. Clinical signs were recorded daily. Blood samples were collected from the surviving mice 30 days post-inoculation (DPI), and 1:25 and 1:200 dilutions of mouse serum were tested for *T. gondii* antibodies with MAT. Tachyzoites or cysts were examined in the lungs or brains of dead or euthanized mice (60 DPI). If tissue cysts or tachyzoites were not found in the mice tissues, homogenized lungs, brain, and heart tissues were subpassaged subcutaneously into a new group of mice, and only one round of mice was inoculated with negative samples.

IHC and transmission electron microscopy (TEM) were performed on mice or cell cultures that were suspected to be infected with *T. gondii*. The primary antibody was rabbit anti-*T. gondii* polyclonal antibody. Cell cultures were fixed with 2.5% glutaraldehyde, embedded in epoxy resin, and then polymerized at 60 °C. Ultrathin sections were cut at 70 nm, stained with uranyl acetate and lead citrate, and then examined at 120 kV using a JEM-1400 Analytical TEM(Tokyo, Japan).

### 2.6. Cell Cultivation and Genotyping

Brain and lung homogenates of *T. gondii*-infected mice were seeded in Vero cell culture flasks (ThermoFisher, Waltham, MA, USA) [2]. DNA was extracted from cell culture-derived tachyzoites. Multiplex PCR was performed on genotype *T. gondii* isolates using 10 PCR-RFLP genetic markers: SAG1, SAG2 (5′-3′SAG2, alt. SAG2), SAG3, BTUB, GRA6, c22-8, c29-2, L358, PK1, and Apico [18]. The virulence genes ROP5 and ROP18 were also typed, as reported previously [19]. Reference *T. gondii* DNA (provided by Dr. Chunlei Su; University of Tennessee, Knoxville, TN, USA) was included in all the batches.

### 2.7. Virulence Evaluation of the T. gondii Strain Isolated from Sheep Using Mice

Fresh *T. gondii* tachyzoites were collected from cell cultures and diluted 10-fold, from 10^−1^ to 10^−5^, to reach an endpoint of <1 tachyzoite. Next, <1, 10^0^, 10^1^, 10^2^, 10^3^, and 10^4^ tachyzoites were inoculated intraperitoneally into four or five Swiss mice for every dilution. Clinical signs, including illness or death, were observed and documented daily. Lung and mesenteric lymph node impression smears of the dead mice were examined for *T. gondii* tachyzoites. At 30 DPI, serum from all the surviving mice was analyzed for antibodies against *T. gondii* using MAT at titers of 1:25 and 1:200. Virulence was evaluated based on the percentage of dead *T. gondii*-positive mice. Mice were euthanized at 60 DPI, brain cysts were counted, and all the tissues were fixed in 10% (*v*/*v*) neutral-buffered formalin.

### 2.8. Statistical Analysis

Statistical analyses were performed using GraphPad Prism 8.0 software (GraphPad Software Inc., San Diego, CA, USA). The data were analyzed using the one-way ANCOVA for the number of brain cysts in *T. gondii*-seropositive mice by time post-infection (at <30 DPI, 30–60 DPI and >60 DPI). The values are expressed as the mean ± SEM, and statistical significance was set at *p* < 0.05.

## 3. Results

### 3.1. T. gondii Infection in Sheep Examined Using MAT and PCR

In this study, 227 sheep serum samples or heart fluids were tested for *T. gondii* antibodies using MAT, and the results indicated that 1.8% (4/227) (95% confidence interval (CI), 0.53–4.60%) of the sheep had anti-*T. gondii* IgG antibodies. The titers of these samples were 1:3200, 1:12,800, 1:3200 (one ewe) and its aborted fetus (1:800). Additionally, two ewes were negative for *T. gondii* IgG antibody, but positive for IgM antibody (Zhumadian). Overall, the seropositivity of *T. gondii* in sheep from Henan and Shandong provinces was 2.0% (4/198) and 0% (0/29), respectively (Table 1, Figure 1).

Molecular diagnostic results showed that 7 of 207 samples were positive for PCR of *T. gondii* DNA (3.4%, 95% CI, 1.51–6.95%) in sheep tissues. These included two myocardial tissues (Jiaozuo and Xuchang) from slaughterhouses, three aborted fetuses (Shangqiu and Zhumadian), and two lambs (Pingdingshan and Xuchang) from veterinary clinics (Table 1).

### 3.2. Isolation of Viable T. gondii from Sheep Myocardial Tissues Using Mouse Bioassay

**Figure 2:**
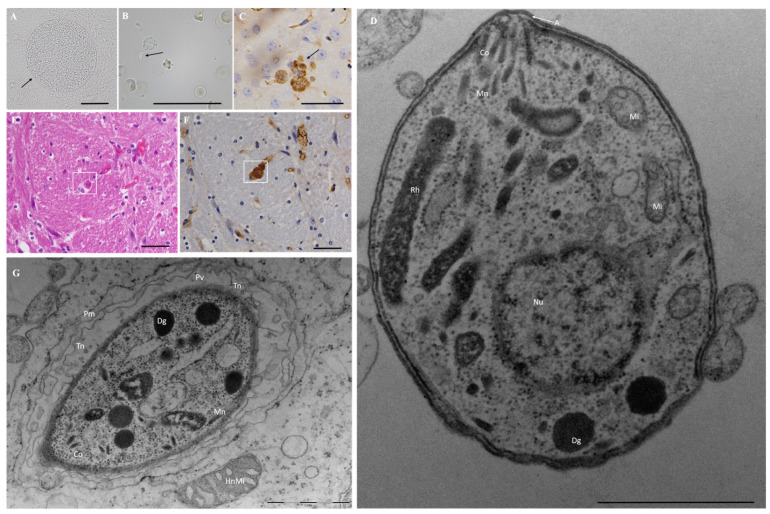
Morphology of *Toxoplasma gondii* TgSheepCHn14. (**A**) *T. gondii* cysts (arrow) were detected in Swiss mouse brain (Tox35-13, M#514), 16 days post-inoculation (DPI), squashed section, unstained, bar = 50 μm; (**B**) *T. gondii* tachyzoites (arrow) were found in the lungs of IFN-γ^−/−^ mouse (Tox35-23, M#476ko), 16 DPI, smear, unstained, bar = 50 μm; (**C**) A cluster of *T. gondii* parasites (arrow) was found in the liver of IFN-γ^−/−^mouse (Tox35-23, M#476ko), 16 DPI, IHC, bar = 50 μm. (**E**,**F**) *T. gondii* parasites (square) were detected in Swiss mouse brain (Tox35-13, M#771), 26 DPI, H&E and IHC, continuous paraffin sections, bar = 50 μm. (**D**,**G**) Tachyzoites from cell culture, the conoid (Co), nucleus (Nu), rhoptries (Rh), micronemes (Mn), dense granules (Dg), apicoplast (A), mitochondrion (Mi), parasitophorous vacuolar membrane (Pm), parasitophorous vacuolar (Pv), tubulovesicular membrane network (Tn), and host cell mitochondrion HnMi were visible, TEM, bar = 1 μm.

Mouse bioassay was performed on four sheep myocardial tissue samples, two of which were seropositive to *T. gondii* (#118 1:3200 Tox35-3; #129 1:12,800 Tox35-4), and the other two were negative for *T. gondii* infection (#47 < 1:25 Tox35-1; #112 < 1:25 Tox35-2) (Supplementary Materials: Figure S1). The results showed that using MAT in the Tox35-4 group, 4/5 mice were seropositive for *T. gondii*. The brain and lungs of mouse M#1216 (Tox35-4) were homogenized and inoculated into the mice of the Tox35-9 and Tox35-13 groups, and the seropositivity of *T. gondii* was 100% (4/4, 4/4) using MAT (≥1:200). Cysts were found in the brain of mouse M#514 (Tox35-13) at 16 DPI (Figure 2A), and *T. gondii* parasites in the brain were verified using hematoxylin and eosin (H&E) and IHC (Figure 2E,F). The brain and lungs of mouse M#764 (Tox35-13) were homogenized and inoculated into the Tox35-15 and Tox35-23 mice groups. The IFN-γ^−/−^ mice (Tox35-15 M#570 and Tox35-23 M#476) died of acute toxoplasmosis (at 22 and 16 DPI, respectively). Tachyzoites were found in the lungs of the mice (Figure 2B), and parasites from the liver tissues were verified using IHC (Figure 2C). 

The growth times of the TgSheepCHn14 strain in Vero cells are summarized in Table 2. The *T. gondii* strain from the brain and lungs of M#764 (Tox35-13) was difficult to propagate in cell cultures (grew after 70 days) and was designated as TgSheepCHn14 (Supplementary Materials: Figure S1). Unfortunately, no viable *T. gondii* strains were isolated from other sheep myocardial tissues.

TEM showed the presence of *T. gondii* TgSheepCHn14 tachyzoites with parasitophorous vacuoles in Vero cells (Figure 2G). The tachyzoites of the TgSheepCHn14 strain were oval or crescent-shaped, 2.6 × 1.8 µm in size (n = 15), and rich in dense granules (Figure 2D). However, they were deficient in amylopectin granules and lipids. 

### 3.3. Identification of the Genotype and Virulence Factor of TgSheepCHn14

The genotype of TgSheepCHn14 was determined using PCR-RFLP with 10 markers, and the results indicated that it belonged to the ToxoDB genotype #3. The ROP18/ROP5 allele combination provides a strong prediction of *T. gondii* strain virulence in mice. The ROP18 and ROP5 loci of the strain were detected, and the allele types were 2/2 (Supplementary Materials: Figure S2). The positive rate and mortality of mice infected with *T. gondii* tachyzoites at different doses are shown in Table 3. None of the mice died of acute infection, but when 10^4^ tachyzoites were inoculated, one mouse died at 37 DPI, and the number of brain cysts from this group of mice was 295.0 ± 285.0. The results showed that this strain was non-lethal to Swiss mice. The numbers ranged from 10 to 1270 cysts per mouse brain, and the number of cysts in the brain tissues of mice increased with an increase in tachyzoite doses, but the difference was not significant (*p* ˃ 0.05). 

**Figure 3:**
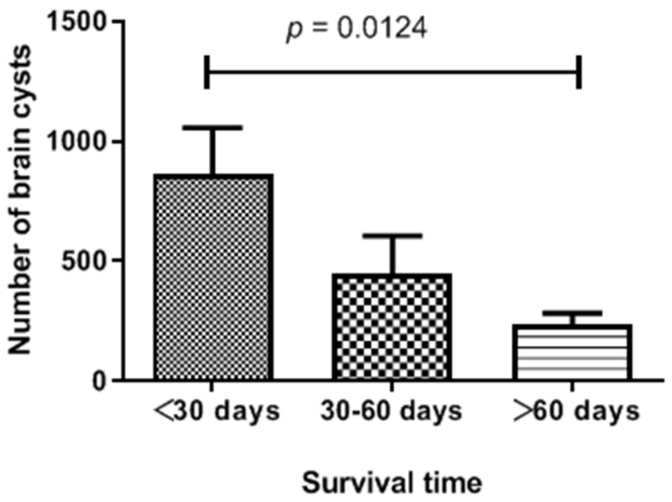
Relationship between the number of *Toxoplasma gondii* brain cysts and survival time in mice (Mean ± SEM).

After completing the statistical analyses, the number of brain cysts in *T. gondii* seropositive mice decreased with time post-infection. Compared with the mice that died before 30 DPI, the number of brain cysts decreased significantly after 60 DPI (*p <* 0.05) (Figure 3).

## 4. Discussion

Ovine toxoplasmosis can cause serious losses in livestock economies worldwide [16,20]. Henan and Shandong provinces are in central China. In this study, the seroprevalence of *T. gondii* in 227 sheep samples from Henan and Shandong provinces was 1.8% (4/227) (Table 1). These results were lower than the seroprevalence of *T. gondii* in sheep in China from 2000 to 2017 [7], and the results were also lower than those in other countries [8]. 

In this study, the seroprevalence of *T. gondii* in sheep from Henan Province was 2.0% (4/198), which was lower than the infection rate of *T. gondii* in sheep (25.3%, 42/166) in Henan Province from 2017 to 2019 [10] (*p* < 0.05). These samples were also subjected to MAT (cut off: 1:100) as the detection method. Prior studies have documented that the seroprevalence in intensively managed sheep farms is lower than that in semi-intensively managed sheep farms [8,21]. All the samples in this study were obtained from intensively managed farms, which may explain the low seroprevalence of *T. gondii*.

In general, the prevalence of *T. gondii* in sheep increases with age, indicating its postnatal transmission. The ingestion of oocysts from the environment is the main route of *T. gondii* infection in sheep [8,22]. However, vertical transmission has also been observed in sheep in many countries [2]. It typically occurs in two ways, namely, exogenous transplacental transmission (by oocysts ingestion) and endogenous transplacental transmission (by reactivation of chronic infection) [2,12,22,23]. Prior studies have suggested that endogenous transplacental transmission occurs infrequently [24,25,26]. A high frequency (13.3%, 4/30) of endogenous transplacental transmission has recently been observed in ewes chronically infected with *T. gondii* [27]. 

In this study, the *T. gondii* antibody titer of the ewe (Shangqiu) was 1:3200, which indicated that it had been exposed to parasites. The *T. gondii* antibody titer of the 2.5-month-old aborted fetus of this ewe was 1:800; further, *T. gondii* DNA was detected in the myocardial tissue of this fetus (Table 1). The syndesmochorial type of placenta in sheep prevents the passage of immunoglobulins; therefore, the antibodies in the fetus may be due to the transmission of parasites across the placenta. Molecular biological evidence from the fetus also supports this hypothesis. 

*Toxoplasma gondii* DNA was in the tissues (lung, heart, liver, or spleen) of two aborted fetuses (4 months) from the same ewe (Zhumadian). The *T. gondii* antibody titers of this ewe and her two aborted fetuses were <1:25, as detected using MAT. As MAT could only detect IgG antibodies against *T. gondii* [15], they were additionally examined for IgM antibodies, and *T. gondii* IgM antibodies were detected in this ewe (Table 1). The two fetuses may have been infected with *T. gondii* through exogenous transplacental transmission, possibly because their mother ingested oocysts during pregnancy.

*Toxoplasma gondii* DNA was found in the tongue and lymph nodes of a one-month-old lamb (Xuchang). *Toxoplasma gondii* antibody titers of this lamb and its dam were <1:25, as detected using MAT. This lamb may have been infected with *T. gondii* via postnatal ingestion of oocysts.

One viable TgSheepCHn14 *T. gondii* strain was successfully isolated from the myocardial tissue of sheep (heart fluid MAT titer was 1:12,800) from a slaughterhouse (Table 1). Prior reports have suggested that MAT titers of 1:100 or higher indicate sheep persistently infected with *T. gondii* [10,16]. The rate of isolation of *T. gondii* increased the antibody titer; it was difficult to isolate from samples with a MAT titer of ˂1:100 [10,28,29,30]. Additionally, no *T. gondii* tissue cysts were found in sheep tissue samples (n = 221 sheep) stained with H&E. Using large samples (50 g) for bioassays may increase the sensitivity of *T. gondii* detection.

The genotype of the TgSheepCHn14 strain belongs to ToxoDB #3 (Type II). Studies on the isolation and genotyping of *T. gondii* from sheep in China are limited. Two viable strains of *T. gondii* were isolated from 304 sheep myocardial tissues between 2014 and 2016, both of which belonged to ToxoDB #9 [9]. Another eleven *T. gondii* strains were isolated from sheep samples from 2017 to 2019, of which seven isolates belonged to ToxoDB#2, and four isolates belonged to ToxoDB #4 [10]. The genotype of the *T. gondii* strain isolated from sheep in Qinghai was ToxoDB #3 [11], which is the same genotype found in this study. ToxoDB #3 strains are predominant in Europe and have also been isolated from sheep in Brazil, the United States, and Ethiopia [8], indicating that this genotype is widespread worldwide. ToxoDB #9 (Chinese 1) is the dominant genotype that is widely prevalent in mainland China [7,31] However, ToxoDB #3 strains have been detected in swine, sparrows, sheep, wild birds, cats, and wallabies from China [11,32,33,34,35,36,37,38]. Studies support the hypothesis that Chinese 1 and Type II *T. gondii* share a common ancestor [39,40,41]. This phenomenon could suggest a continuum between Type II and Chinese 1, as well as the circulation of this genotype strain through the Silk Road.

ROP18 and ROP5 are the main determinants of *T. gondii* virulence [19,42,43,44,45]. The ROP18 and ROP5 alleles of TgSheepCHn14 (2/2) and the strain were non-lethal in mice, which was consistent with the prediction of prior studies [19,46]. The TgSheepCHn14 strain was genotype II, and the number of *T. gondii* cysts in the brain of mice was 414.2 ± 74.6 (n = 26), ranging from 10 to 1270 cysts. In prior studies, the *T. gondii* cyst load of the type II strain was relatively high in the central nervous system of mice [47]. This may be related to the immune status of the host and the inoculation dose [48]. 

More importantly, we found that TgSheepCHn14 was smaller than other strains (RH, Me49, VEG, TgMonkeyCHn1, and TgRooCHn4), and it was deficient in amylopectin granules [2,38,49]. It took a long time to grow in vitro and was difficult to propagate in cell culture. Further, in vivo, the number of TgSheepCHn14 cysts in mice brain tissues decreased with time post-infection (Figure 3). This indicates that some cysts may have been eliminated by the host. However, the underlying mechanisms remain unknown. IL-21 and IL-15 can reduce the number of *T. gondii* cysts in the brain [50]. Cattle are considered poor hosts for *T. gondii* because they have more effective immune responses for eliminating *T. gondii* from their tissues [51,52]. Overall, this newly isolated strain (TgSheepCHn14) exhibited distinctive features that distinguished it from other strains. The limitation of this study was that the samples were not from planned collections by Epi_Info 7 software; instead, these samples were scattered, some samples were collected in slaughterhouses, and some arrived in the laboratory for pathology diagnostics. 

## 5. Conclusions

Here, vertical transmission of *T. gondii* occurred in two of the three pairs of sheep (ewes and pups). To our knowledge, this was the first survey to report *T. gondii* antibody and *T. gondii* DNA in aborted fetuses from China, and it indicated that *T. gondii* could maintain in sheep herds. One viable *T. gondii* strain (TgSheepCHn14, ToxoDB genotype #3) was isolated from the myocardial tissues of sheep from slaughterhouses. The seroprevalence of *T. gondii* in sheep has recently decreased in China. This may be due to the improved intensive management of farms. However, sheep are also exposed to the zoonotic parasites, and mutton may serve as a potential source of human *T. gondii* infections. 

## Figures and Tables

**Table 1:** Prevalence and isolation of a viable *Toxoplasma gondii* strain from sheep.

Province	Location ^a^	Source	Number of Samples	Number, MAT Titers ^b^	Positive Number/Tested Number by PCR ^c^	Isolation by Mice Group ^d^	Additional Information (Positive Tissues by PCR)
Henan	I: Sanmenxia	Vet	2 lambs	2, <1:25	0/2	nd	-
	II: Jiaozuo	Sh	135 hearts	133, <1:25 2, ≥1:3200	1/135	1/4 TgSheepCHn14	#129 lamb, 8-month, (H)
		Vet	2 lambs	2, <1:25	0/2	nd	-
	III: Zhoukou	Vet	1 lamb	1, <1:25	0/1	nd	-
		Sh	9 hearts	9, <1:25	nd	nd	-
	IV: Xinxiang	Sh	30 hearts	30, <1:25	0/30	nd	-
	V: Pingdingshan	Vet	1 lamb	1, <1:25	1/1	nd	1 month, diarrhea, (H)
	VI: Luoyang	Vet	1 lamb	1, <1:25	0/1	nd	-
	VII: Xuchang	Vet	1 lamb *	1, <1:25	1/1	nd	Streptococcicosis 1 month, (T, LN)
			1 dam serum *	1, <1:25	nd	nd	-
		Sh	7 hearts and legs	7, <1:25	1/2, 5 nd	nd	#5, 10-month, (H, Lm)
	VIII: Shangqiu	Vet	1 aborted fetus *	1, 1:800	1/1	nd	2.5-month, autolysis Brucellosis (H)
			1 dam serum *	1, 1:3200	nd	nd	Only serum
	IX: Zhumadian	Vet	2 aborted fetuses *	2, <1:25	2/2	nd	4-month, malnutrition #1: (H, Li, Lu); #2: (Sp, Lu)
			4 dam sera *	4, <1:25	nd	nd	Dam#1, #3: IgM positive ^e^
Shandong	X: Heze	Sh	29 hearts	29, <1:25	0/29	nd	-
Total				1.8% (4/227)	3.4% (7/207)		

^a^: Figure 1 shows sampling cities. ^b^: MAT: modified agglutination test. ^c^: PCR: polymerase chain reaction. ^d^: Number of positive groups/number of inoculated sheep samples. ^e^: The *T. gondii* IgM antibody for Dam#1 and Dam#3 was positive using *Toxoplasma gondii* test paper, two aborted fetus were from Dam#1. nd: experiment not performed. Vet: Veterinary clinic; Sh: Slaughterhouse; H: heart; T: tongue; LN: lymph nodes; Lm: leg muscle; Li: liver; Lu: lung; Sp: spleen. *: The asterisk designates the relationship between lamb and its mother.

**Table 2:** TgSheepCHn14 strain growth times in Vero cells.

Mice No.	Samples	Seeding Date	Date When Many Tachyzoites Were Observed	Growth Time (Days)
Tox35-9, M#156, Swiss mouse	Brain	24 June 2020	26 September 2020	94
Tox35-13, M#764, Swiss mouse	Brain, lungs	15 July 2020	23 September 2020	70
Tox35-15, M#570, IFN-γ knockout mouse	Ascitic fluid	6 September 2020	20 September 2020	14
Tox35-23, M#476, IFN-γ knockout mouse	Lungs	15 November 2020	30 November 2020	15

**Table 3:** Pathogenicity of the *Toxoplasma gondii* TgSheepCHn14 strain isolated from sheep on Swiss mice using intraperitoneal (double check).

Concentration of Tachyzoites	10^0^	10^1^	10^2^	10^3^	10^4^
TgSheepCHn14 (Tachyzoites derived from Tox35-15 M#570 IFN-γ^−/−^ ascites)					
*T. gondii* positive rate	0% (0/5) ^a^	0% (0/5)	0% (0/5)	0% (0/5)	80% (4/5)
Mortality%	0	0	0	0	0 (0/4) ^b^
Survival days/Number of infections	˃60/0	˃60/0	˃60/0	˃60/0	˃60/4
Number of brain cysts	-	-	-	-	197.5 ± 89.6
TgSheepCHn14 (Tachyzoites were derived from Tox35-23 M#476 IFN-γ^−/−^ cell culture)					
*T. gondii* positive rate	0% (0/4) ^a^	0% (0/4)	75% (3/4)	100% (4/4)	100% (4/4)
Mortality%	0	0	0 (0/3)	0 (0/4)	33.3 (1/4) ^b^
Survival days/Number of infections	˃60/0	˃60/0	˃60/3	˃60/4	˃60/3, 37/1
Number of brain cysts	-	-	10.0	60.0	295.0 ± 285.0

^a^: Amount of infections/number of inoculations. ^b^: Number of natural deaths after infection/number of infections.

## Data Availability

The datasets used and/or analyzed in this study are available from the corresponding author upon reasonable request.

## Supplementary Materials

The following supporting information can be downloaded at: https://www.mdpi.com/article/10.3390/pathogens12060827/s1, Figure S1: Flow chart for sheep infected *Toxoplasma gondii* assay procedure; Figure S2: Genotyping of *Toxoplasma gondii* isolated strains from sheep; Table S1: Summary of viable *Toxoplasma gondii* isolates from animals and human in China (n = 169); Table S2: Raw data about the number of *Toxoplasma gondii* TgSheepCHn14 brain cysts and survival time in mice.

## Abbreviations

MAT: modified agglutination test; PCR: polymerase chain reaction; H&E: hematoxylin and eosin; IHC: immunohistochemistry; TEM: transmission electron microscopy; DPI: days post-inoculation.

## References

[1] Dubey J.P. (2022). Toxoplasmosis of Animals and Humans.

[2] Dubey J.P. (2010). Toxoplasmosis of Animals and Humans.

[3] Liesenfeld O., Montoya J. (2004). Toxoplasmosis. Lancet.

[4] Dubey J.P., Casey S.J., Zajac A.M., Wildeus S.A., Lindsay D.S., Verma S.K., Oliveira S., Kwok O.C., Su C. (2014). Isolation and genetic characterization of *Toxoplasma gondii* from alpaca (*Vicugna pacos*) and sheep (*Ovis aries*). Trop. Anim. Health. Prod..

[5] Dubey J.P., Verma S.K., Ferreira L.R., Oliveira S., Cassinelli A.B., Ying Y., Kwok O.C.H., Tuo W., Chiesa O.A., Jones J.L. (2014). Detection and survival of *Toxoplasma gondii* in milk and cheese from experimentally infected goats. J. Food Prot..

[6] Tonouhewa A.B.N., Akpo Y., Sessou P., Adoligbe C., Yessinou E., Hounmanou Y.G., Assogba M.N., Youssao I., Farougou S. (2017). *Toxoplasma gondii* infection in meat animals from Africa: Systematic review and meta-analysis of seroepidemiological studies. Vet. World.

[7] Dong H., Su R., Lu Y., Wang M., Liu J., Jian F., Yang Y. (2018). Prevalence, risk factors, and genotypes of *Toxoplasma gondii* in food animals and humans (2000–2017) from China. Front. Microbiol..

[8] Dubey J., Murata F., Cerqueira-Cézar C., Kwok O., Su C. (2020). Economic and public health importance of *Toxoplasma gondii* infections in sheep: 2009–2020. Vet. Parasitol..

[9] Yang Y., Feng Y., Yao Q., Wang Y., Lu Y., Liang H., Zhu X., Zhang L. (2017). Seroprevalence, isolation, genotyping, and pathogenicity of *Toxoplasma gondii* strains from sheep in China. Front. Microbiol..

[10] Jiang H.-H., Huang S.-Y., Zhou D.-H., Zhang X.-X., Su C., Deng S.-Z., Zhu X.-Q. (2013). Genetic characterization of *Toxoplasma gondii* from pigs from different localities in China by PCR-RFLP. Parasites Vectors.

[11] Zhou P., Zhang H., Lin R.-Q., Zhang D.-L., Song H.-Q., Su C., Zhu X.-Q. (2009). Genetic characterization of *Toxoplasma gondii* isolates from China. Parasitol. Int..

[12] Hide G. (2016). Role of vertical transmission of *Toxoplasma gondii* in prevalence of infection. Expert Rev. Anti. Infect. Ther..

[13] Pinto-Ferreira F., Caldart E.T., Pasquali A.K.S., Mitsuka-Bregano R., Freire R.L., Navarro I.T. (2019). Patterns of transmission and sources of infection in outbreaks of human toxoplasmosis. Emerg. Infect. Dis..

[14] Edwards J.F., Dubey J. (2013). *Toxoplasma gondii* abortion storm in sheep on a Texas farm and isolation of mouse virulent atypical genotype *T. gondii* from an aborted lamb from a chronically infected ewe. Vet. Parasitol..

[15] Dubey J.P., Desmonts G. (1987). Serological responses of equids fed *Toxoplasma gondii* oocysts. Equine Vet. J..

[16] Dubey J.P. (2009). Toxoplasmosis in sheep—The last 20 years. Vet. Parasitol..

[17] Schares G., Herrmann D., Beckert A., Hosseininejad M., Pantchev N., Vrhovec M.G., Conraths F. (2008). Characterization of a repetitive DNA fragment in *Hammondia hammondi* and its utility for the specific differentiation of *H. hammondi* from *Toxoplasma gondii* by PCR. Mol. Cell. Probes.

[18] Su C., Shwab E.K., Zhou P., Zhu X.Q., Dubey J.P. (2009). Moving towards an integrated approach to molecular detection and identification of *Toxoplasma gondii*. Parasitology.

[19] Shwab E.K., Jiang T., Pena H., Gennari S.M., Dubey J.P., Su C. (2016). The ROP18 and ROP5 gene allele types are highly predictive of virulence in mice across globally distributed strains of *Toxoplasma gondii*. Int. J. Parasitol..

[20] Buxton D., Maley S.W., Wright S.E., Rodger S., Bartley P., Innes E.A. (2007). *Toxoplasma gondii* and ovine toxoplasmosis: New aspects of an old story. Vet. Parasitol..

[21] Wang C., Qiu J., Gao J., Liu L., Liu Q., Yan C., Zhu X. (2011). Seroprevalence of *Toxoplasma gondii* infection in sheep and goats in northeastern China. Small Rumin. Res..

[22] Innes E.A., Bartley P.M., Buxton D., Katzer F. (2009). Ovine toxoplasmosis. Parasitology.

[23] Carlier Y., Truyens C., Deloron P., Peyron F. (2012). Congenital parasitic infections: A review. Acta Trop..

[24] Andrade G.M.Q., Vasconcelos-Santos D.V., Carellos E.V.M., Romanelli R.M.C., Vitor R.W.A., Carneiro A.C.A.V., Januario J.N. (2009). Congenital toxoplasmosis from a chronically infected woman with reactivation of retinochoroiditis during pregnancy. J. Pediatr..

[25] Buxton D., Rodger S., Maley S., Wright S. (2006). Toxoplasmosis: The possibility of vertical transmission. Small Rumin. Res..

[26] Rodger S.M., Maley S.W., Wright S.E., Mackellar A., Wesley F., Sales J., Buxton D. (2006). Role of endogenous trans-placental transmission in toxoplasmosis in sheep. Vet. Rec..

[27] Costa F.T., Nogueira D.B., Oliveira M.A., Silva S.S., Silva R.F., Sarmento W.F., Azevedo S.S., Gennari S.M., Pena H.F., Brasil A.W. (2021). Vertical transmission of *Toxoplasma gondii* in naturally infected ewes in the semiarid region of Brazil. Comp. Immunol. Microbiol. Infect. Dis..

[28] Dubey J.P., Laurin E., Kwowk O.C.H. (2016). Validation of the modified agglutination test for the detection of *Toxoplasma gondii* in free-range chickens by using cat and mouse bioassay. Parasitology.

[29] Dubey J., Sundar N., Hill D., Velmurugan G., Bandini L., Kwok O., Majumdar D., Su C. (2008). High prevalence and abundant atypical genotypes of *Toxoplasma gondii* isolated from lambs destined for human consumption in the USA. Int. J. Parasitol..

[30] Dubey J.P., Thulliez P., Weigel R.M., Andrews C.D., Lind P., Powell E.C. (1995). Sensitivity and specificity of various serologic tests for detection of *Toxoplasma gondii* infection in naturally infected sows. Am. J. Veter-Res..

[31] Fu X.Y., Feng Y.J., Liang H.D., Yang Y.R. (2015). Genotypes and pathogenesis of *Toxoplasma gondii* isolates in China. Chin. J. Zoonoses.

[32] Cong W., Huang S.-Y., Zhou N.-H., Zhang X.-X., Zhang N.-Z., Zhao Q., Zhu X.-Q. (2013). Prevalence and Genetic Characterization of *Toxoplasma gondii* in House Sparrows (*Passer domesticus*) in Lanzhou, China. Korean J. Parasitol..

[33] Huang S.Y., Cong W., Zhou P., Zhou D.H., Wu S.M., Xu M.J., Zou F.C., Song H.Q., Zhu X.Q. (2012). First report of genotyping of *Toxoplasma gondii* isolates from wild birds in China. J. Parasitol..

[34] Jiang N., Su R., Jian F., Su C., Zhang L., Jiang Y., Yang Y. (2020). *Toxoplasma gondii* in lambs of China: Heart juice serology, isolation and genotyping. Int. J. Food Microbiol..

[35] Tian Y.-M., Huang S.-Y., Miao Q., Jiang H.-H., Yang J.-F., Su C., Zhu X.-Q., Zou F.-C. (2014). Genetic characterization of *Toxoplasma gondii* from cats in Yunnan Province, Southwestern China. Parasites Vectors.

[36] Wang D., Liu Y., Jiang T., Zhang G., Yuan G., He J., Su C., Na Yang N. (2016). Seroprevalence and genotypes of *Toxoplasma gondii* isolated from pigs intended for human consumption in Liaoning province, northeastern China. Parasites Vectors.

[37] Yang L., Xin S., Zhu N., Li J., Su C., Yang Y. (2023). Two viable *Toxoplasma gondii* isolates from red-necked wallaby (*Macropus rufogriseus*) and red kangaroo (*M. rufus*). Parasitol. Int..

[38] Yang L., Ren H., Zhu N., Mao G., Li J., Su C., Jiang Y., Yang Y. (2023). Epidemiology and isolation of viable *Toxoplasma gondii strain* from macropods. Heliyon.

[39] Chaichan P., Mercier A., Galal L., Mahittikorn A., Ariey F., Morand S., Boumédiène F., Udonsom R., Hamidovic A., Murat J.B. (2017). Geographical distribution of *Toxoplasma gondii* genotypes in Asia: A link with neighboring continents. Infect Genet. Evol..

[40] Bertranpetit E., Jombart T., Paradis E., Pena H., Dubey J., Su C., Mercier A., Devillard S., Ajzenberg D. (2016). Phyloge-ography of *Toxoplasma gondii* points to a South American origin. Infect. Genet. Evol..

[41] Lorenzi H., Khan A., Behnke M.S., Namasivayam S., Swapna L.S., Hadjithomas M., Karamycheva S., Pinney D., Brunk B.P., Ajioka J.W. (2016). Local admixture of amplified and diversified secreted pathogenesis determinants shapes mosaic *Toxoplasma gondii* genomes. Nat. Commun..

[42] Boothroyd J.C., Dubremetz J.-F. (2008). Kiss and spit: The dual roles of *Toxoplasma rhoptries*. Nat. Rev. Genet..

[43] Bradley P.J., Sibley L.D. (2007). Rhoptries: An arsenal of secreted virulence factors. Curr. Opin. Microbiol..

[44] Niedelman W., Gold D.A., Rosowski E., Sprokholt J.K., Lim D., Arenas A., Melo M., Spooner E., Yaffe M.B., Saeij J.P.J. (2012). The rhoptry proteins ROP18 and ROP5 mediate *Toxoplasma gondii* evasion of the Murine, but not the human, interferon-gamma response. PLoS Pathog..

[45] Saeij J.P.J., Boyle J.P., Coller S., Taylor S., Sibley L.D., Brooke-Powell E.T., Ajioka J.W., Boothroyd J.C. (2006). Polymorphic secreted kinases are key virulence factors in toxoplasmosis. Science.

[46] Khan A., Fux B., Su C., Dubey J.P., Darde M.L., Ajioka J.W., Rosenthal B.M., Sibley L.D. (2007). Recent transcontinental sweep of *Toxoplasma gondii* driven by a single monomorphic chromosome. Proc. Natl. Acad. Sci. USA.

[47] Gatkowska J., Wieczorek M., Dziadek B., Dzitko K., Dlugonska H. (2012). Behavioral changes in mice caused by *Toxoplasma gondii* invasion of brain. Parasitol. Res..

[48] Dubey J.P., Ferreira L.R., Martins J., McLeod R. (2012). Oral oocyst-induced mouse model of toxoplasmosis: Effect of infection with Toxoplasma gondii strains of different genotypes, dose, and mouse strains (transgenic, out-bred, in-bred) on pathogenesis and mortality. Parasitology.

[49] Xin S., Jiang N., Yang L., Zhu N., Huang W., Li J., Zhang L., Su C., Yang Y. (2022). Isolation, genotyping and virulence determination of a *Toxoplasma gondii* strain from non-human primate from China. Transbound. Emerg. Dis..

[50] Li Z.Y., Chen J., Petersen E., Zhou D.H., Huang S.Y., Song H.Q., Zhu X.Q. (2014). Synergy of mIL-21 and mIL-15 in enhancing DNA vaccine efficacy against acute and chronic *Toxoplasma gondii* infection in mice. Vaccine.

[51] Dubey J.P., Murata F.H.A., Cerqueira-Cezar C.K., Kwok O.C.H., Yang Y.R. (2020). Public health significance of *Toxoplasma gondii* infections in cattle: 2009–2020. J. Parasitol..

[52] Esteban-Redondo I., Innes E.A. (1997). *Toxoplasma gondii* infection in sheep and cattle. Comp. Immunol. Microbiol. Infect. Dis..

