# Supplementary material for: Low Prevalence of Toxoplasma gondii in Sheep and Isolation of a Viable Strain from Edible Mutton from Central China
Source: Pathogens. 2023 Jun 14;12(6):827. doi: 10.3390/pathogens12060827 (PMC10302579; doi:10.3390/pathogens12060827)
Supplement: Supplementary file 1 [file pathogens-12-00827-s001.zip › pathogens-2385158-supplementary.pdf]

Supplementary materials

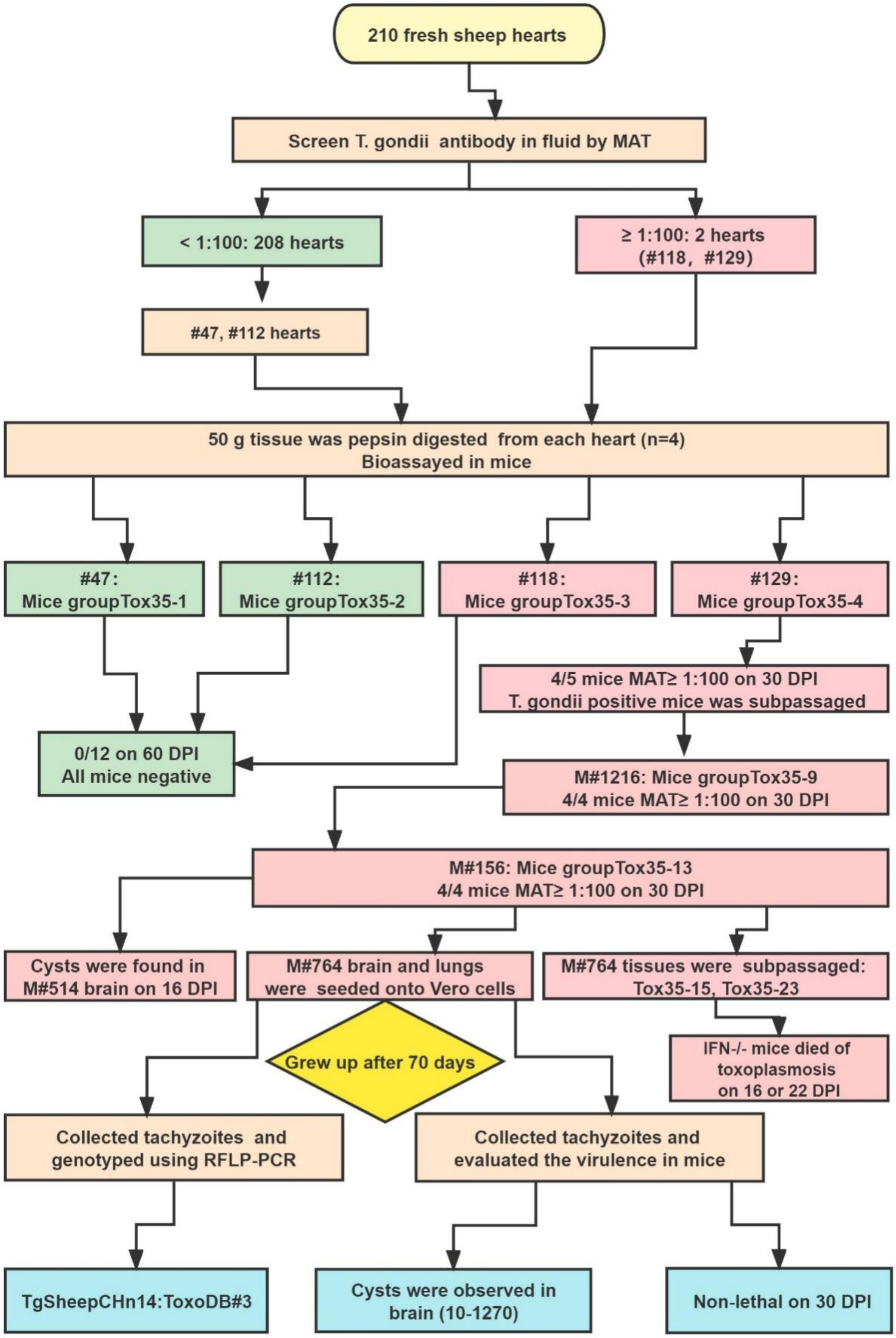

Figure S1. Flow chart for sheep infected *Toxoplasma gondii* assay procedure.

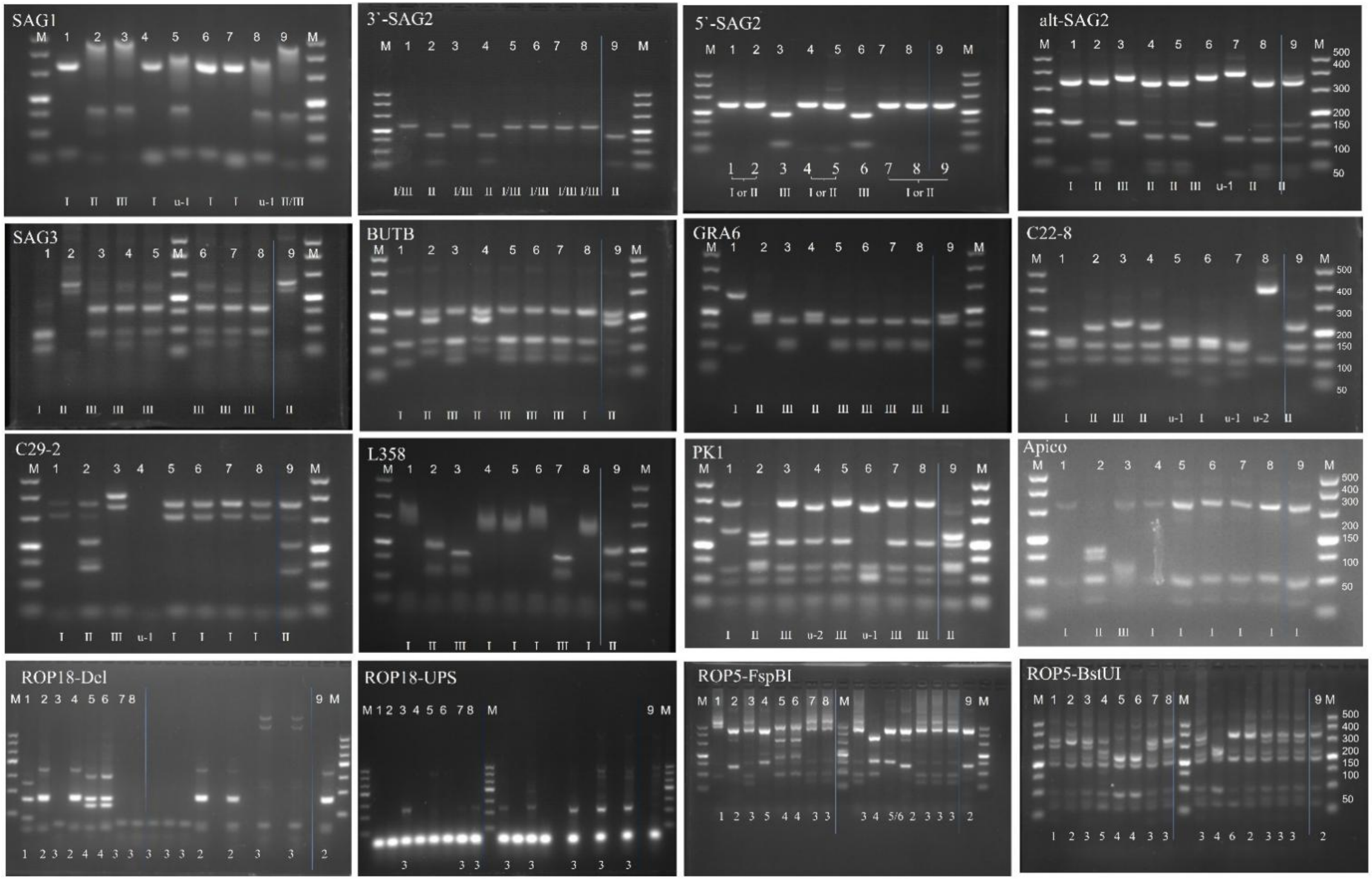

Figure S2. Genotyping of *Toxoplasma gondii* isolated strains from sheep.

1:GT1, 2: PTG, 3: CTG, 4: TgCgCaI, 5: MAS, 6: TgCatBr5, 7: TgCatBr64, 8: TgToucan (TgRsCr1), 9: TgSheepCHn14, M: markers.

Table S1 Summary of viable *Toxoplasma gondii* isolates from animals and human in China (n = 169)

Province	species	No. of animls	Seroprevalence	Bioassay of tissues[b]			Genotype ToxoDB #	References
			Test, cut-off titer (%)	Number	Positive	Strain ID		
Beijing	Stray cat	64	MAT, 20 (58)	23	11	TgCatBj1-11	All #9	[1]
Anhui (22), Hubei (23), Guangdong (20), Shanxi (24)	cat	211	ND	89	14	TgCtys1,2 TgCtwh1-8, TgCtgd1,2 TgCtsx1,2	12 #9, 2 #205	[2]
ND	Human cat	ND	ND	ND	23	TgHuZSE, TgHuZS2, TgHuAh1-2, TgCtwh9-12,14,19 TgCtxz1-8, TgCtsd1-5	15 #9, 2 #10, 1 #1, 1 #204, 4 #205	[3]
Guizhou (23+17), Anhui (24), Hubei (13)	Cat,Pig, Chicken,vole	77	ND	B	11	TgCtgy1-5, TgCtgy1-4 TgCksz1, TgVowh1	10 #9, 1 #205	[4]
Guangdong	Market cat	34	MAT, 40(79)	27	17	TgCtPRC1-17	2#18, 15#9	[5]
Guangdong	Human, Pig, Cat, Sheep	ND	ND	ND	17	SH, CN,NT, TgC1-TgC8 GYS,PYS,ZC,ZS,ZS1,QHO	3 #10, 1 #3, 12 #9, 1 # MIX	[6]
Shanghai	rabbit	77	MAT,25(23)	18	1	SHR	SAG2, SAG3, GRA6, BTUB All type III	[7]
Liaoning	pig	2063	MAT,25(11.3)	233	23	TgPigCn3-2, 3-12, 5-5,5-6,5-7, 8-16, 8-18,10-34,14-56,19-24, 22-10,27-8, 29-5, 33-6,40-10, 58-14, 61-11,69-2, 69-14,77-2,80-11,80-25,80-27	1#3, 1#3 or #1, 13#9	[8]
Kunming	feline feces	115	Morphology, (4)	ND	1	KM isolate	type II	[9]
Guizhou	Pig, cat	70+19	ELISA, (49+12)	ND	5	TGGZ1-3(pig), TGGZ4-5(cat)	All #9	[10]
Jiangsu	human fetus pigs	ND	ND	ND	3	KS, YZ-1, YZ-2	1 #10, 2#9	[11-12]
Henan [c], Zhejiang	Stray cat, pet cat	42	MAT, 100(50)	42	8+1=9	TgCatCHn1-3, TgCatCZg1-5 TgCatCHn4 (feces)	6 #9, 1 #1, 1 #2, 1 #17	[13]
Henan	Hospital cat	28	MAT,25(7)	8,	1	TgCatCHn5	#9, 1	[14]
Henan	Sheep	840	MAT,25(20.7)	26	2	TgSheepHn1-2	All #9	[15]
Henan	Sheep	166	MAT,100(25)	67	11	TgSheepHn3-13	7 #2, 4 #4	[16]
Henan	Sheep	224	MAT,100(1.8)	4	1	TgSheepHn14	#3	This study
Henan	Kangaroo	4	MAT,25(75)	4	1	TgRooCHn1	#292	[17]
Henan	Kangaroo	3	MAT,25(67)	3	2	TgRooCHn2-3	#3, #2	[18]
Henan	Kangaroo	26	MAT,25(50)	6	1	TgRooCHn4	#3	[19]
Henan	Red panda	14	MAT,25(14)	8	1	TgRedpandaCHn1	#20	[20]
Henan	Serval	1	MAT,25(100)	1	1	TgServalCHn1	#20	[21]

Henan	Tiger	10	MAT,25(80)	6	2	TgTigerCHn1-2	All #9	[22]
Henan	Tiger	4	MAT,25(100)	4	2	TgTigerCHn3-4	#20, #2	[23]
Henan	Caracal	2	MAT,25(100)	2	1	TgCaracalCHn1	#2	[24]
Henan	Caracal	1	MAT,25(100)	1	1	TgCaracalCHn2	#5	Ren HJ et al., unpublished
Henan	White spoonbills	7	MAT,4(29)	2	1	TgSpoonbillCHn1	#2	[25]
Henan	Monkey	15	MAT,8(47)	9	1	TgMonkeyCHn1	Mix	[26]
Henan	Monkey	13	MAT,8(62)	9	1	TgMonkeyCHn2	#9	Yang LL et al., unpublished
Henan	Monkey	9	MAT,8(22)	3	1	TgMonkeyCHn3	#6	Ma YH et al., unpublished
Henan	Cheetah	6	MAT,25(100)	6	2	TgCheetahCHn1-2	#319, #9	Zhu NP et al., unpublished
Henan	Fur seal	4	MAT,25(50)	4	1	TgfursealCHn1	#5	Mao GH et al., unpublished
Total	110 #9, 12 #2, 7 #205, 6 #10, 6 #3, 4 #4, 3 #20, 2 #1, 2 #18, 2 #5, 1 #6, 1 #204, 1 #17, 1 #292, 1 #319							

ND: experiment not done;

MAT: modified agglutination test;

ELISA: enzyme-linked immunosorbent assay.

Table S2 Raw data about the number of *Toxoplasma gondii* TgSheepCHn14 brain cysts and survival time in mice.

Survival time / Brain cysts	＜30 days	30-60 days	＞60 days
1	1190	760	400
2	70	160	130
3	1270	190	360
4	500	680	180
5	1140		370
6	1000		90
7			60
8			10
9			110
10			450
11			40
12			260
13			350
14			420
15			570
16			10
M±SE	862±194	448±158	238±45

References

1. Qian, W.; Wang, H.; Su, C.; Shan, D.; Cui, X.; Yang, N.; Lv, C.; Liu, Q. Isolation and characterization of *Toxoplasma gondii* strains from stray cats revealed a single genotype in Beijing, China. *Vet Parasitol*. **2012**, *187(3-4)*, 408–413.
2. Chen, Z.W.; Gao, J.M.; Huo, X.X.; Wang, L.; Yu, L.; Halm-Lai, F.; Xu, Y.H.; Song, W.J.; Hide, G.; Shen, J.L.; Lun, Z.R. Genotyping of *Toxoplasma gondii* isolates from cats in different geographic regions of China. *Vet Parasitol.* **2011**, *183(1-2)*, 166–70.
3. Wang, L.; Chen, H.; Liu, D.; Huo, X.; Gao, J.; Song, X.; Xu, X.; Huang, K.; Liu, W.; Wang, Y.; Lu, F.; Lun, Z.R.; Luo, Q.; Wang, X.; Shen, J. Genotypes and mouse virulence of *Toxoplasma gondii* isolates from animals and humans in China. *PLoS One*. **2013**, *8(1)*, e53483.
4. Wang, L.; Cheng, H.W.; Huang, K.Q.; Xu, Y.H.; Li, Y.N.; Du, J.; Yu, L.; Luo, Q.L.; Wei, W.; Jiang, L.; Shen, J.L. *Toxoplasma gondii* prevalence in food animals and rodents in different regions of China: isolation, genotyping and mouse pathogenicity. *Parasit Vectors*. **2013**, *6*, 273.
5. Dubey, J.P.; Zhu, X.Q.; Sundar, N.; Zhang, H.; Kwok, O.C.; Su, C. Genetic and biologic characterization of *Toxoplasma gondii* isolates of cats from China. *Vet Parasitol*. **2007**, *145(3-4)*, 352–356.
6. Zhou, P.; Zhang, H.; Lin, R.Q.; Zhang, D.L.; Song, H.Q.; Su, C.; Zhu, X.Q. Genetic characterization of *Toxoplasma gondii* isolates from China. *Parasitol Int*. **2009**, *58(2)*, 193–195.
7. Zhou, Y.; Zhang, H.; Cao, J.; Gong, H.; Zhou, J. Isolation and genotyping of *Toxoplasma gondii* from domestic rabbits in China to reveal the prevalence of type III strains. *Vet Parasitol*. **2013**, *193(1-3)*, 270–276.
8. Wang, D.; Liu, Y.; Jiang, T.; Zhang, G.; Yuan, G.; He, J.; Su, C.; Yang, N. Seroprevalence and genotypes of *Toxoplasma gondii* isolated from pigs intended for human consumption in Liaoning province, northeastern China. *Parasit Vectors*. **2016**, *9*, 248.
9. Liang, Y.; Chen, J.; Meng, Y.; Zou, F.; Hu, J.; Esch, G.W. Occurrence and genetic characterization of GRA6 and SAG2 from *Toxoplasma gondii* oocysts in cat feces, Kunming, China. *Southeast Asian J Trop Med Public Health*. **2016**, *47(6)*, 1134–1142.
10. Li, Y.N.; Nie, X.; Peng, Q.Y.; Mu, X.Q.; Zhang, M.; Tian, M.Y.; Min, S.J. Seroprevalence and genotype of *Toxoplasma gondii* in pigs, dogs and cats from Guizhou province, Southwest China. *Parasit Vectors*. **2015**, *8*, 214.
11. Hou, Z.; Zhou, Y.; Liu, D.; Su, S.; Zhao, Z.; Xu, J.; Tao, J. Genotyping and virulence analysis of *Toxoplasma*

gondii isolates from a dead human fetus and dead pigs in Jiangsu province, Eastern China. *Acta Parasitol*. **2018**, *63(2)*, 397–411.

12. Zhang, W.; Liu, J.S.; Ma, Y.W.; Lu, S. *Toxoplasma gondii* isolated from a dead deformed fetus. *Chinese Journal of Zoonoses*, **1987**, *3*, 26 (in Chinese).
13. Yang, Y.; Ying, Y.; Verma, S.K.; Cassinelli, A,B.; Kwok, O,C.; Liang, H.; Pradhan, A.K.; Zhu, X.Q.; Su, C.; Dubey, J.P. Isolation and genetic characterization of viable *Toxoplasma gondii* from tissues and feces of cats from the central region of China. *Vet Parasitol*. **2015**, *211(3-4)*, 283–288.
14. Yang, Y.R.; Feng, Y.J.; Lu, Y.Y.; Dong, H.; Li, T.Y.; Jiang, Y,B.; Zhu, X.Q.; Zhang, L.X. Antibody detection, isolation, genotyping, and virulence of *Toxoplasma gondii* in captive felids from China. *Front Microbiol*. **2017**, *8*, 1414.
15. Yang, Y.; Feng, Y.; Yao, Q.; Wang, Y.; Lu, Y.; Liang, H.; Zhu, X.; Zhang, L. Seroprevalence, isolation, genotyping, and pathogenicity of *Toxoplasma gondii* strains from sheep in China. *Front Microbiol*. **2017**, *8*, 136.
16. Jiang, N.; Su, R.; Jian, F.; Su, C.; Zhang, L.; Jiang, Y.; Yang, Y. *Toxoplasma gondii* in lambs of China: heart juice serology, isolation and genotyping. *Int J Food Microbiol*. **2020**, *322*, 108563.
17. Su, R.; Dong, H.; Li, T.; Jiang, Y.; Yuan, Z.; Su, C.; Zhang, L.; Yang, Y. *Toxoplasma gondii* in four captive kangaroos (*Macropus* spp.) in China: Isolation of a strain of a new genotype from an eastern grey kangaroo (*Macropus giganteus*). *Int J Parasitol Parasites Wildl*. **2019**, *8*, 234–239.
18. Yang, L.; Xin, S.; Zhu, N.; Li, J.; Su, C.; Yang, Y. Two viable *Toxoplasma gondii* isolates from red-necked wallaby (*Macropus rufogriseus*) and red kangaroo (*M. rufus*). *Parasitol Int*. **2023**, *92*, 102687.
19. Yang, L.; Ren, H.; Zhu, N.; Mao, G.; Li, J.; Su, C.; Jiang, Y.; Yang, Y. Epidemiology and isolation of viable *Toxoplasma gondii* strain from macropods. *Heliyon*. **2023**, *9(3)*, e13960.
20. Yang, Y.; Dong, H.; Su, R.; Li, T.; Jiang, N.; Su, C.; Zhang, L. Evidence of red panda as an intermediate host of *Toxoplasma gondii* and *Sarcocystis* species. *Int J Parasitol Parasites Wildl*. **2019**, *8*, 188–191.
21. Dong, H.; Su, R.; Li, T.; Su, C.; Zhang, L.; Yang, Y. Isolation, genotyping and pathogenicity of a *Toxoplasma gondii* strain isolated from a Serval (*Leptailurus serval*) in China. *Transbound Emerg Dis*. **2019**, *66(4)*, 1796–1802.
22. Yang, Y.; Dong, H.; Su, R.; Jiang, N.; Li, T.; Su, C.; Yuan, Z.; Zhang, L. Direct evidence of an extra-intestinal cycle of *Toxoplasma gondii* in tigers (*Panthera tigris*) by isolation of viable strains. *Emerg. Microbes Infect*. **2019**, *8(1)*, 1550–1552.

23. Ren, H.; Yang, L.; Zhu, N.; Li, J.; Su, C.; Jiang, Y.; Yang, Y. Additional evidence of tigers (*Panthera tigris altaica*) as intermediate hosts for *Toxoplasma gondii* through the isolation of viable strains. *Int J Parasitol Parasites Wildl*. **2022**, *19*, 330–335.
24. Jiang, N.; Xin, S.; Li, J.; Su, C.; Zhang, L.; Yang, Y. Isolation and characterization of *Toxoplasma gondii* from captive caracals (*Caracal caracal*). *Int J Parasitol Parasites Wildl*. **2020**, *13*, 196–201.
25. Yang, Y.; Jiang, N.; Xin, S.; Zhang, L. *Toxoplasma gondii* infection in white spoonbills (*Platalea leucorodia*) from Henan Province, China. *Emerg Microbes Infect*. **2020**, *9(1)*, 2619–2621.
26. Xin, S.; Jiang, N.; Yang, L.; Zhu, N.; Huang, W.; Li, J.; Zhang, L.; Su, C.; Yang, Y. Isolation, genotyping and virulence determination of a *Toxoplasma gondii* strain from non-human primate from China. *Transbound Emerg Dis*. **2022**, *69(2)*, 919–925.
